# Supplementary material for: ENT Residents Benefit from a Structured Operation Planning Approach in the Training of Functional Endoscopic Sinus Surgery
Source: Medicina (Kaunas). 2021 Oct 4;57(10):1062. doi: 10.3390/medicina57101062 (PMC8541081; doi:10.3390/medicina57101062)
Supplement: Supplementary file 1 [file medicina-57-01062-s001.zip › medicina-1390737-supplementary.pdf]

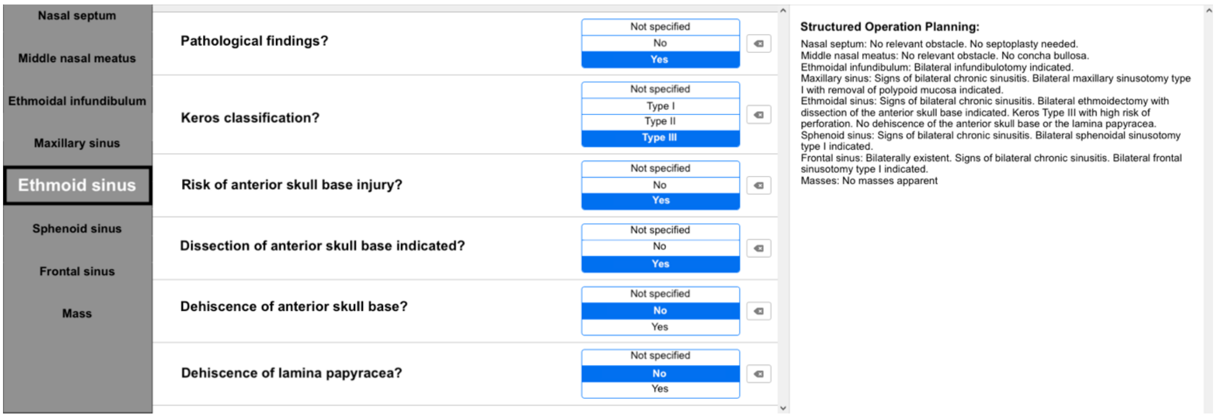
Nasal septum
Middle nasal meatus
Ethmoidal infundibulum
Maxillary sinus
Ethmoid sinus
Sphenoid sinus
Frontal sinus
Mass
Pathological findings?
Not specified
No
Yes
Keros classification?
Not specified
Type I
Type II
Type III
Risk of anterior skull base injury?
Not specified
No
Yes
Dissection of anterior skull base indicated?
Not specified
No
Yes
Dehiscence of anterior skull base?
Not specified
No
Yes
Dehiscence of lamina papyracea?
Not specified
No
Yes
Structured Operation Planning:
Nasal septum: No relevant obstacle. No septoplasty needed.
Middle nasal meatus: No relevant obstacle. No concha bullosa.
Ethmoidal infundibulum: Bilateral infundibulotomy indicated.
Maxillary sinus: Signs of bilateral chronic sinusitis. Bilateral maxillary sinusotomy type I with removal of polypoid mucosa indicated.
Ethmoidal sinus: Signs of bilateral chronic sinusitis. Bilateral ethmoidectomy with dissection of the anterior skull base indicated. Keros Type III with high risk of perforation. No dehiscence of the anterior skull base or the lamina papyracea.
Sphenoid sinus: Signs of bilateral chronic sinusitis. Bilateral sphenoidal sinusotomy type I indicated.
Frontal sinus: Bilaterally existent. Signs of bilateral chronic sinusitis. Bilateral frontal sinusotomy type I indicated.
Masses: No masses apparent
